# Sociosexual and Communication Deficits after Traumatic Injury to the Developing Murine Brain

**DOI:** 10.1371/journal.pone.0103386

**Published:** 2014-08-08

**Authors:** Bridgette D. Semple, Linda J. Noble-Haeusslein, Yong Jun Kwon, Pingdewinde N. Sam, A. Matt Gibson, Sarah Grissom, Sienna Brown, Zahra Adahman, Christopher A. Hollingsworth, Alexander Kwakye, Kayleen Gimlin, Elisabeth A. Wilde, Gerri Hanten, Harvey S. Levin, A. Katrin Schenk

**Affiliations:** 1 Department of Neurological Surgery, University of California San Francisco, San Francisco, California, United States of America; 2 Department of Medicine (Royal Melbourne Hospital), Melbourne Brain Centre, University of Melbourne, Parkville, Victoria, Australia; 3 Department of Physical Therapy and Rehabilitation, University of California San Francisco, San Francisco, California, United States of America; 4 Department of Physics, Randolph College, Lynchburg, Virginia, United States of America; 5 San Francisco State University, San Francisco, California, United States of America; 6 Physical Medicine and Rehabilitation Alliance of Baylor College of Medicine and the University of Texas-Houston Medical School, Houston, Texas, United States of America; 7 Michael E. DeBakey Veterans Affairs Medical Center, Houston, Texas, United States of America; Texas Christian University, United States of America

## Abstract

Despite the life-long implications of social and communication dysfunction after pediatric traumatic brain injury, there is a poor understanding of these deficits in terms of their developmental trajectory and underlying mechanisms. In a well-characterized murine model of pediatric brain injury, we recently demonstrated that pronounced deficits in social interactions emerge across maturation to adulthood after injury at postnatal day (p) 21, approximating a toddler-aged child. Extending these findings, we here hypothesized that these social deficits are dependent upon brain maturation at the time of injury, and coincide with abnormal sociosexual behaviors and communication. Age-dependent vulnerability of the developing brain to social deficits was addressed by comparing behavioral and neuroanatomical outcomes in mice injured at either a pediatric age (p21) or during adolescence (p35). Sociosexual behaviors including social investigation and mounting were evaluated in a resident-intruder paradigm at adulthood. These outcomes were complemented by assays of urine scent marking and ultrasonic vocalizations as indices of social communication. We provide evidence of sociosexual deficits after brain injury at p21, which manifest as reduced mounting behavior and scent marking towards an unfamiliar female at adulthood. In contrast, with the exception of the loss of social recognition in a three-chamber social approach task, mice that received TBI at adolescence were remarkably resilient to social deficits at adulthood. Increased emission of ultrasonic vocalizations (USVs) as well as preferential emission of high frequency USVs after injury was dependent upon both the stimulus and prior social experience. Contrary to the hypothesis that changes in white matter volume may underlie social dysfunction, injury at both p21 and p35 resulted in a similar degree of atrophy of the corpus callosum by adulthood. However, loss of hippocampal tissue was greater after p21 compared to p35 injury, suggesting that a longer period of lesion progression or differences in the kinetics of secondary pathogenesis after p21 injury may contribute to observed behavioral differences. Together, these findings indicate vulnerability of the developing brain to social dysfunction, and suggest that a younger age-at-insult results in poorer social and sociosexual outcomes.

## Introduction

Survivors of childhood traumatic brain injury (TBI) have an elevated risk of long-term social dysfunction [1], [2], with substantial implications for reintegration into society and quality of life [3]. In children, severe TBI is associated with poorer relationships such as low peer acceptance and high levels of rejection and victimization in the classroom [4], [5]. In addition, brain injury at a young age is consistently predictive of poorer language and non-verbal communication [6], [7], [8], which may complicate the ongoing development of social skills [9]. These consequences persist up to 20 years after severe TBI during childhood [10], [11], [12], and compromise many areas of language competence including syntax, semantics and pragmatics [13]. Clinical studies have identified an association between deficits in social communication and the degree of white matter damage [14], thought to result from diffuse axonal injury which disrupts connectivity between regions of the distributed ‘social brain’ network [15]. Further, a younger age-at-insult has been highlighted as a risk factor for social dysfunction [6], leading to the hypothesis that impairments after childhood TBI may reflect a disturbance in brain development and the failure to acquire age-appropriate skills [16], [17].

Previously, we have demonstrated an age-dependent emergence of social deficits after TBI to the mouse at postnatal day (p) 21, approximating a toddler-aged child [18]. Specifically, male brain-injured mice showed a reduction in social investigation, sociability and social novelty preferences compared to sham control mice when tested at adulthood, but not when tested earlier at adolescence, suggesting that the full extent of deficits may not be evident until the associated skills reach maturity [19]. However, it remains unclear whether the developing brain is more vulnerable to such social dysfunction due to its immaturity – in other words, whether these deficits are dependent upon the age-at-insult, as has been suggested from recent clinical findings [20]. Furthermore, despite the life-long implications of social and communication dysfunction after TBI, assays for social communication have not yet been explored in the context of experimental TBI.

Rodents communicate primarily by using olfactory and acoustic signals [21]. Behaviorally, these signals are comprised of physical contact in the form of social investigation, scent marking, and both audible and ultrasonic vocalizations (USVs). Mice deposit pheromone-containing urinary traces in a context-specific manner to mark territories, attract potential mates, and communicate information about health and dominance status [22], [23], [24], [25]. A reduction in scent marking has been observed in several mouse strains characterized by low sociability, and quantification of scent marking is now employed as an assay of the communication deficits typically seen in autism models [26], [27], [28], [29]. We hypothesized that this assay may also be valuable for detecting changes in social behavior after experimental TBI.

An alternative mode of social communication between rodents is by USVs. Robust USVs are typically emitted by male mice in response to the presence of females or female urine [30], [31], and may correlate with particular courtship behaviors such as mounting and social interest [21], [32]. Although our understanding of USVs is far from complete, playback and devocalization experiments demonstrate that USVs convey important information between individuals, and there is increasing acceptance that changes in USV production reflect communication abnormalities [21], [33], [34]. Although USV's have not yet been explored in the context of experimental TBI, changes have been reported in several genetic models of autism, which reflect a spectrum of social deficits similar to those seen in TBI survivors [26], [29], [35], [36], [37].

This is the first comprehensive assessment of social behavior and sociosexual communication in a pediatric model of TBI. Here we consider age at time of injury as a determinant of long-term outcomes, using a battery of behavioral assays including novel metrics (scent marking and USVs) that have yet to be tested in the context of TBI. We further determine if the structure of the corpus callosum, which mediates the majority of inter-hemispheric connections between cortices, is vulnerable to pediatric TBI, and if that vulnerability is predictive of long-term deficits in post-traumatic social outcomes. Lastly, we evaluated hippocampal volume loss as an indicator of lesion progression.

## Methods

### Ethics Statement

All experiments were approved by the University of California San Francisco (UCSF) Institutional Animal Care and Use Committee (approval number #AN100120), and conducted in strict compliance with the NIH Guidelines for the Care and Use of Laboratory Animals to minimize any pain or suffering of the animals.

### Animals

**Figure 1:**
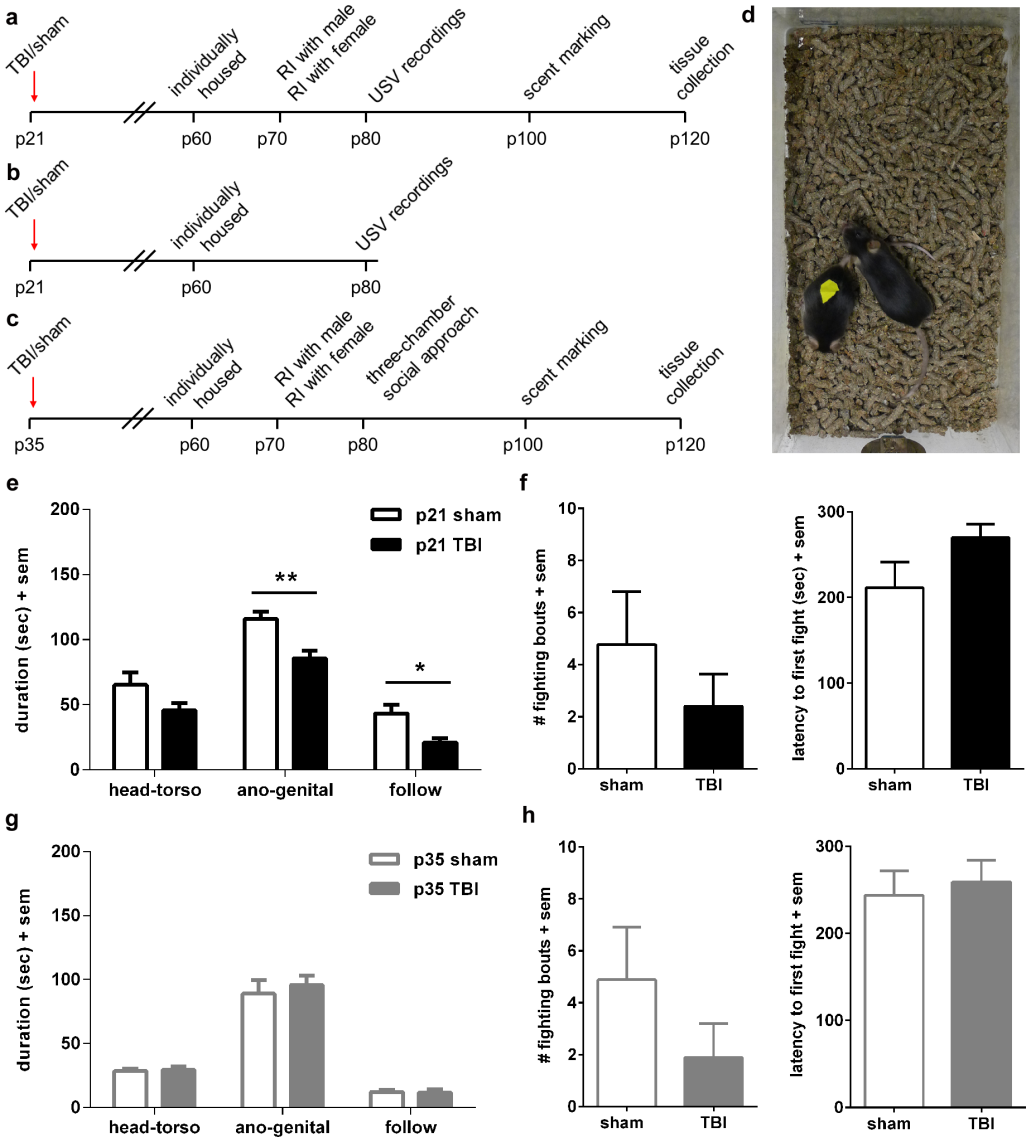
Reduced social investigation at adulthood after pediatric but not adolescent TBI. Experimental timelines illustrate the timing of behavioral assessments; Cohort 1 (a) consisted of n = 10/group after TBI or sham-operation at p21; Cohort 2 (b) was n = 7–8/group, also after TBI or sham at p21; Cohort 3 (c) consisted of mice that received TBI or sham-operation at p35 (adolescence; n = 9/group). Investigative and interactive behaviors by sham and TBI mice, at adulthood after p21 or p35 injury, were quantified after addition of a novel male mouse in the resident-intruder (RI) paradigm (d). Brain-injured mice that received TBI at p21 (e) spent less time engaged in social investigative behaviors including ano-genital sniffing and following (**p<0.01, *p<0.05). Antagonistic behaviors (f), quantified as the number of fighting bouts and latency to first fight, were not different between the injury groups. After injury at p35 (g, h), sham and TBI mice also showed similar investigative behaviors towards a novel male mouse.

Male C57Bl/6J pups at post-natal day 17 with an accompanying lactating mother were purchased from The Jackson Laboratory (Bar Harbor, ME) and housed in the Laboratory Animal Resource Center at UCSF. Mice were weaned at p21 and group-housed (4–5/cage) with littermates unless otherwise stated. Standard rodent chow and tap water were available *ad libitum*, and the room was maintained on a 12 hour light/dark cycle at approximately 20°C. A total of 53 mice were used in this study, in three experimental cohorts: cohort 1 (n = 10 sham and n = 10 TBI at p21; Figure 1a), cohort 2 (n = 7 sham and n = 8 TBI at p21; Figure 1b) and cohort 3 (n = 9 sham and n = 9 TBI at p35; Figure 1c).

### Controlled cortical impact (CCI) model

At p21 (described henceforth as ‘pediatric’) or p35 (corresponding to ‘adolescence’) ±1 day, mice were anesthetized with 1.25% or 2% 2,2,2-tribromoethanol in isotonic saline respectively, administered i.p. at 0.02 ml/g body weight. Mice from each litter were randomly allocated for either TBI or sham operation. After positioning of the head in a stereotaxic frame (David Kopf Instruments, Tujunga, CA), the skull was exposed by reflection of soft tissues and a craniotomy was performed over the left parietal lobe. Mice that were randomly allocated as ‘TBI’ were then positioned beneath the injury device (Custom Design and Fabrication, eCCI-6.3) and subjected to a moderate-severe controlled cortical impact injury as previously described [19], [38], [39]. The injury was generated at 4.5 m/sec velocity and 1.7 mm depth of penetration, for a sustained depression of 150 msec, using a 3.0 mm convex impactor tip. Mice were maintained on a 37°C water-circulating heating pad throughout surgery and recovery. Following impact, the scalp was closed with sutures and each animal administered ∼0.5 ml isotonic saline s.c. to prevent post-operative dehydration. Sham-operated mice underwent identical surgical procedures, including craniotomy, without receiving the cortical impact. Following surgery, mice were group-housed with littermates (4–5/cage) and provided with standard bedding supplemented with a cardboard dome house and nestlet square. Weights were monitored weekly post-surgery, and were comparable between sham and TBI mice in all cohorts (figure S1).

### Behavioral Assessments

Behavioral procedures were conducted and analyzed by an investigator blinded to treatment (sham or TBI) by random ear tag assignment after surgery. It has previously been reported that social investigation and vocalizations are enhanced following a period of social isolation [40], therefore, test mice were separated into individual cages one week prior to behavioral testing, at early adulthood (∼p60). This design was based upon evidence that social behaviors may change across developmental age [41], [42], [43], [44], therefore both cohorts were assessed at the same age. Testing conditions were identical to those previously described for social behavior assessments [19]. ‘Stimulus’ animals were naïve age-matched male and female C57Bl/6J mice, purchased at the same time from Jackson Laboratory and group-housed by sex on the opposite side of the same room. Each test mouse (sham or TBI) encountered each stimulus mouse on one occasion only, ensuring that the stimulus mouse was always novel.

### Resident-intruder task for social investigation

The resident-intruder task examines the social response of the test mouse to a novel stimulus mouse. A stimulus mouse (male or female) was presented into the home cage of the test mouse and behaviors were video-recorded during a 5 min session as previously described [19], [45] Social behaviors of the test mouse towards the stimulus mouse were scored blinded from videos using Stopwatch+^©^ (Center for Behavioral Neuroscience, Georgia State University), and categorized based on published literature [46], [47] as follows: head-torso sniff, ano-genital sniff, and following (defined as the test mouse following the stimulus mouse nose-to-tail). In addition, fighting (including biting and wrestling) and mounting behaviors were recorded during encounters with a stimulus male or female, respectively. Mounting attempts were identified as the test male approaching the stimulus mouse from behind, grabbing with its forepaws, and exhibiting rapid pelvic thrusting motions [48]. The number and duration of behaviors were recorded, as well as latency to the first occurrence. The absence of a behavior during the test period was assigned a latency of 300 sec maximum. Each test mouse encountered one novel male and one novel female stimulus mouse in this paradigm, on separate days.

### Three-chamber social approach task

The three-chamber paradigm allows for the evaluation of social affiliation and social recognition in mice [49]. The task was performed here after injury at p35, as previously described in detail after injury at p21 [19]. In brief, three consecutive stages of 10 min each allow for (1) habituation in an empty 3-chambered box; (2) a choice between an empty cup and a cup containing a novel male stimulus mouse; and (3) choice between a second novel stimulus mouse and the first, now familiar mouse. Stages 2 and 3 are measures of the test mouse's preference for sociability and social recognition, respectively. Data are expressed as time spent in each chamber (% of total time).

### Ultrasonic Vocalizations

Ultrasonic vocalizations (USVs) were quantified as a potential measure of social communication after TBI or sham-operation at p21. Recording of USV's was performed at ∼p80, one week following the resident-intruder task (Cohort 1) or housing isolation (Cohort 2). Mice were placed into an open-topped plexiglass enclosure, which was either circular (for female bedding stimulus; 8.5 cm diameter) or rectangular (for addition of a male or female stimulus mouse; 7×15 cm; see figure S2). This enclosure was placed either on the bench top (Cohort 1) or in a sound-attenuating chamber (Cohort 2; Med Associates Inc, St Albans, VT) and the test mouse was allowed a 30 min habituation period. Vocalizations were then recorded from an Avisoft UltraSoundGate CM16/CMPA microphone (1–180,000 Hz; Berlin, Germany) suspended a fixed distance above the enclosure. Recordings were collected using a National Instruments data acquisition board and processed in MATLAB v7.10.1 (MathWorks, Natick, MA). After a 30 sec ‘baseline’ recording period (no stimulus), the stimulus was added to the enclosure and vocalizations were recorded for the following 120 sec. As the number of calls during baseline measurements was negligible (0–3 calls per test) and did not differ between treatment groups, analyses presented are for the 120 sec when the stimulus was present. USV's were recorded during three different paradigms: (1) addition of a novel male mouse; (2) addition of a novel female mouse; and (3) addition of female bedding, a 3–4 cm diameter clump of soiled bedding collected fresh from a cage of group-housed, stimulus female mice [50]. ‘Cohort 1’ consisted of mice which had previously undergone the resident-intruder test and were later used for scent marking and histology experiments (n = 10/group; figure 1a). ‘Cohort 2’ was a second group of mice who had no other behavioral assessments prior to USV recordings (n = 7–8/group; figure 1b). Mice that produced fewer than 5 calls during the recording period following addition of the stimulus were excluded as ‘non-responders.’

Sound files were filtered and de-noised using custom built software in MATLAB v7.10.1. Individual USV calls were detected using an automated detection algorithm as previously described [36]. Call detection was then checked by visual inspection of the spectrogram and calculated sound pressure envelope. False detections (scratches and other noises mistaken for calls) were removed and missed calls were added to the call tally. All analyses were performed independently by two investigators blinded to injury condition. When two mice were present during a test (i.e. addition of a male or female stimulus mouse), the total calls emitted were analyzed together. Calls emitted during encounters with a female stimulus were presumed to originate from the male test subject; although females have the capacity to vocalize in the ultrasonic range, previous studies have demonstrated that they rarely do so when paired with a male [51], [52], [53], [54]. Parameters that were analyzed included total number of calls, number of calls with median frequency above a set threshold (75 kHz), call duration, median call frequency, latency to the first call, and call burst characteristics. The median frequency of a call was calculated using the technique outlined in [36]. Histograms of the square root of the number of calls within mean median frequency bins of 2.886 kHz were plotted to determine the frequency threshold of 75 kHz. Call bursts were verified by a trained observer by quantifying the inter-call intervals (ICI), the time between the end of one call and the start of the next. ICI's were log-transformed to examine the temporal distribution of calls [36], and a burst interval threshold was identified. Once bursts were identified by implementing the burst interval threshold, the mean burst duration, number of calls contained within bursts, and fraction of calls in bursts was calculated.

### Open Field and Scent Marking

Scent marking in response to a female stimulus was assessed at adulthood after injury at p21 and p35, as a measure of sociosexual communication and recognition [22]. A standard open field arena (40.64×40.64 cm; Hamilton-Kinder, Julian, CA) was lined with a sheet of specialized paper (Strathmore Drawing Paper Premium 400 series, Strathmore Artist Paper, Neenah, WI). To obtain baseline measurements, mice were allowed free exploration of the arena for 10 minutes, during which time interfaced Motor Monitor software was used to calculate parameters including total distance traveled (a measure of general activity) and relative time spent in the center versus the periphery (a measure of anxiety) [39]. Baseline scent marking and defecation was also quantified. For the test phase, the open field arena was thoroughly cleaned and then lined with fresh paper, and a novel adult female was gently placed inside an inverted, custom-made stainless steel barred cup (6.5×15 cm) in the center of the arena. A test mouse (sham or TBI) was then placed individually into this open field space and allowed free exploration for 20 minutes.

After the test, both mice were returned to their respective home cages and the scent-marked paper was allowed to dry overnight at room temperature. Scent-marked papers were treated with Ninhydrin spray reagent (0.2% in ethanol; Sigma Aldrich, St. Louis, MO) and allowed to dry for 24–48 h, which allowed for the visualization of urine traces as purple marks. Quantification of scent marking was adapted from that previously described [22], [26]. A 10 mm×10 mm grid was overlaid onto the paper, and every square or part thereof which contained a scent mark was counted as one unit. The total number of scent marks within a 20 cm diameter circle around the female was quantified. The numbers of fecal boli were also recorded as a measure of defecation.

### Tissue collection and preparation

Anesthetized mice were perfused transcardially with ice-cold saline followed by 4% paraformaldehyde (PFA) in 0.1M phosphate-buffered saline. Brains were removed whole and post-fixed overnight in 4% PFA, then transferred into a 30% sucrose solution for 72 h. Brains were then embedded in Neg50 (Richard-Allan Scientific, Thermo Fisher Scientific, Kalamazoo, MI) for storage at −80°C. Coronal sections spanning the entire cortex were cut at 40 µm on a Leica cryostat and collected serially onto Fisherbrand Superfrost* Plus slides (Thermo Fisher Scientific). As scent marking behavior may be dependent upon urinary output, and bladder function may be altered after TBI [55], bladders were collected for post-mortem examination prior to perfusion. Dissected bladders were expressed to empty residual urine, blotted dry and weighed, and measurements of length (major axis) and width (minor axis) were used to estimate volume based upon the shape of a prolate spheroid of major axis L and minor axis W, as previously described (V = 4/3πL[W/2]^2^) [56], [57].

### Volumetric analysis of white matter and hippocampal loss

Volumetric estimation of the corpus callosum and dorsal hippocampus was performed on cresyl violet-stained sections collected at adulthood after injury or sham-operation at p21 or p35. The unbiased Cavalieri method was conducted with Stereo Investigator software (MicroBrightField v10.21.1, Williston, VT), using a Nikon E600W microscope configured with a motorized stage, MAC 5000 controller and Retiga 2000R color digital camera (QImaging, Surrey, BC, Canada). Systematic random sampling was applied using a sampling interval of 6, to measure 16–20 sections spanning the corpus callosum and 6–8 sections spanning the hippocampus, anteriorly to posteriorly. Hippocampal measurements were confined to the dorsal hemispheres which contained the impacted region. The inferior boundary was defined anteriorly by a horizontal line from the most ventral point of the 3rd ventricle at the midline, and posteriorly by the most dorsal point of the posterior commissure. Regions ipsilateral and contralateral to the injury site were quantified separately, using a 4× objective and grid size of 50 µm. Brains in which >2 sections could not be analyzed due to suboptimal quality were excluded. Thus volumetric analyses were conducted for a subset of mice (n = 5 sham at p21; n = 5 TBI at p21; n = 6 sham at p35; n = 9 TBI at p35). All measurements were conducted by investigators blinded to treatment group, with the Gundersen mean coefficient of error (m = 0) for individual estimates maintained ≤0.08. Group means are expressed as estimated volume (mm^3^).

### Statistical analysis

Data were analyzed as appropriate using GraphPad Prism version 4.03 or SPSS version 19.0.0. Analysis of variance (ANOVA) with repeated measures (RM) was used to analyze behaviors observed during the resident-intruder tasks, with a between-subjects factor of injury and a within-subjects factor of behavior. Interactions between factors are reported if statistically significant. Bonferroni tests were used for post-hoc comparisons, reported graphically as *p<0.05, **p<0.01, ***p<0.001 and ****p<0.0001. Unpaired t-tests were employed to compare total investigative time between groups, or Mann-Whitney tests for non-parametric data that failed normality tests. RM ANOVAs were also used to compare volumetric loss in the corpus callosum and hippocampus, with a between-subjects factor of injury and a within-subjects factor of region (hemisphere), followed by Sidak's post-hoc analyses. Fisher's exact tests (2-sided) were used to compare the percentage of tissue loss relative to sham controls between age cohorts. USV call characteristics were analyzed by RM ANOVAs with a between-subjects factor of injury and a within-subjects factor of stimulus (female, bedding or male), followed by one-way ANOVA's to specifically identify injury-dependent differences within each stimulus condition. For USV call derived qualities such as the latency to call, call duration and call median frequency, only mice that responded to all three stimuli were included in an RM ANOVA analysis (Cohort 1: n = 6 sham and n = 6 TBI; Cohort 2: n = 6 sham and n = 4 TBI), and these data are presented in the results and graphically. To confirm these findings in a larger cohort, we also performed one-way ANOVAs within each stimulus condition and included all mice that responded to that particular stimulus (for Cohort 1: n = 9, 10 and 6 per group for female, female bedding and male stimuli, respectively; for Cohort 2: n = 6–8, 5–7 and 5–6 for female, female bedding and male stimuli, respectively). This analysis consistently confirmed the one-way ANOVAs from the RM data unless stated. F and t-statistics are reported to 2 decimal places, and p-values >0.0001 reported to 3 decimal places. A probability level of p<0.05 was considered statistically significant.

## Results

### Reduced social investigation towards male conspecifics at adulthood is unique to pediatric TBI

Social investigation was examined by introducing a male stimulus mouse into the home cage of the test male mouse (sham or TBI), at adulthood after injury at either p21 or p35 (figure 1a, c). After surgery at p21, sham-operated mice spent a total of 224.5±18.1 sec investigating the intruding male over a 5 min period, compared to TBI mice that spent 32% less time engaged in social investigation (152.1±8.51 sec; unpaired t-test, t_18_ = 3.75, p = 0.002). The duration of different investigative behaviors within this five minute encounter was also analyzed (figure 1e), demonstrating a predominance of ano-genital sniffing over interest in the head-torso region, and following (2-way RM ANOVA effect of behavior F_2, 34_ = 90.66, p<0.0001; effect of injury F_2, 17_ = 14.05, p = 0.002). Post-hoc analyses revealed specific reductions by TBI mice in the time spent sniffing the ano-genital region of the stimulus male (p<0.01), as well as the duration engaged in following behavior (p<0.05), compared to sham-operated mice. Fighting between the test and stimulus mice was not significantly different between sham and TBI mice in terms of the number of occurrences (t_18_ = 1.02, p = 0.320; figure 1f). However, TBI mice tended to show a longer latency to engage in fighting behavior compared to sham animals; although this did not reach statistical significance (t_18_ = 1.77, p = 0.095), it may reflect fewer TBI mice engaging in fighting during the test period (40% compared to 60% of sham mice).

In a separate experiment, mice were assessed in the resident-intruder task at adulthood after TBI or sham-operation at adolescence (p35) (figure 1g). The total time spent investigating the stimulus mouse during the 5 min session was comparable between sham and TBI mice (129.9±13.4 sec, versus 137.7±8.60 sec; unpaired t-test, t_16_ = 0.43, p = 0.672). Further analyses revealed that both sham and TBI mice spent the majority of this time conducting ano-genital sniffing, with considerably less time dedicated to sniffing the head-torso region or following (2-way RM ANOVA effect of behavior F_2, 32_ = 143.10, p<0.0001). Antagonistic behavior was also similar, with 44% of sham-operated and 33% of brain-injured mice engaging in fighting with the stimulus mouse during the testing period (figure 1h; Mann-Whitney test p = 0.4679) and a comparable latency to the first fight (t_16_ = 0.71, p = 0.686).

### Changes in sociosexual behavior at adulthood after pediatric but not adolescent TBI

**Figure 2:**
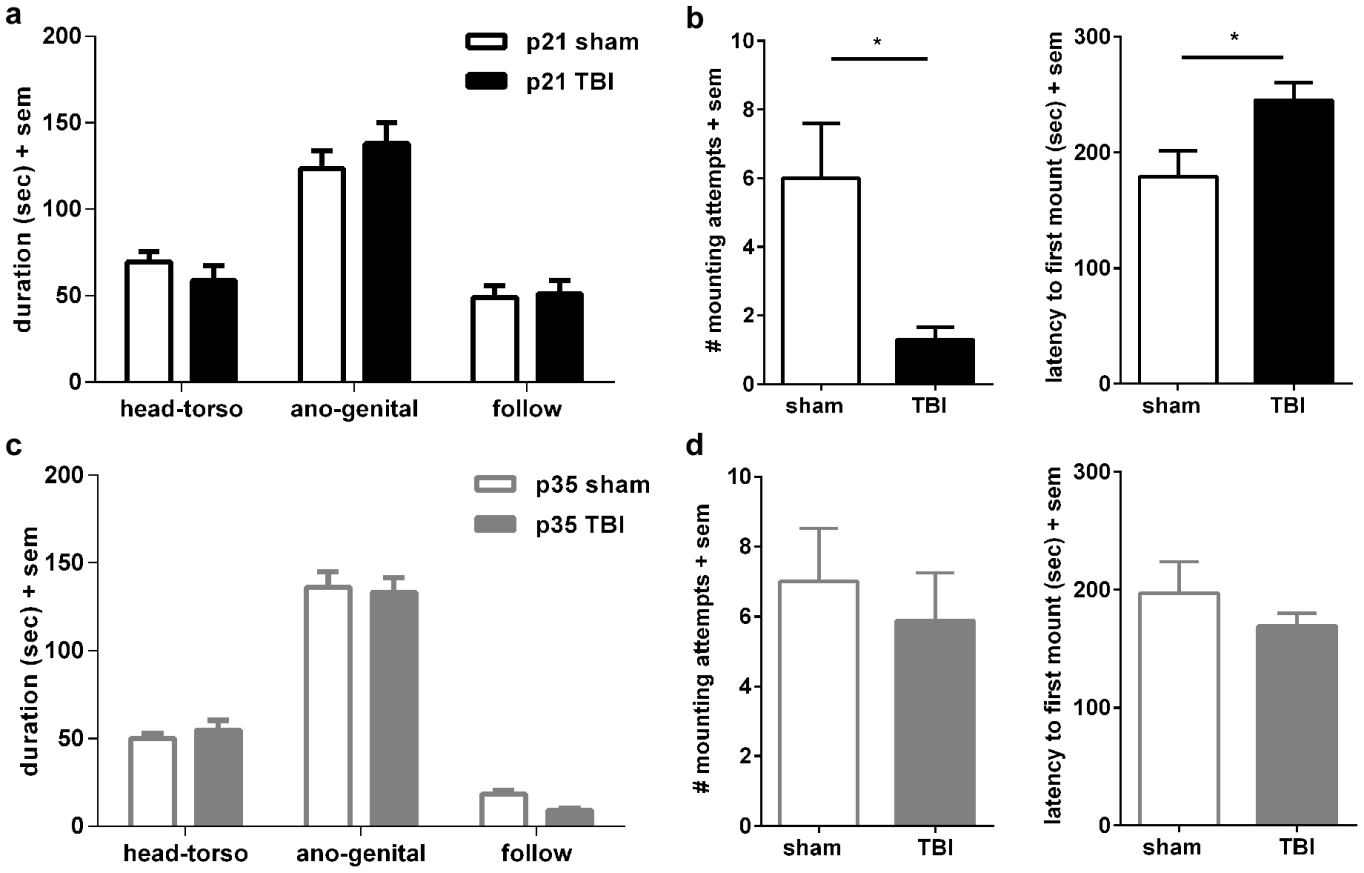
Changes in sociosexual investigation at adulthood after pediatric but not adolescent TBI. Investigative behaviors were quantified at adulthood after addition of a novel female mouse, after injury or sham-operation at p21 or p35. Sham and TBI mice injured at p21 showed similar investigative behaviors overall (a). However, sexual behavior was markedly reduced (b), with TBI mice initiating fewer mounting attempts, and a delayed latency to the first attempt (*p<0.05). After injury at p35 (c), both sham and TBI mice showed similar investigative behaviors towards a novel female mouse. Sexual behavior, quantified as the number of mounting attempts and latency to first attempt, were also comparable in sham and TBI mice after injury at adolescence (d).

The resident-intruder paradigm was also employed to examine social investigation in a sociosexual context, by introducing a *female* stimulus mouse into the home cage of the test male mouse (sham or TBI; figure 2). At adulthood after surgery at p21, in contrast to male-to-male interactions, both sham-operated and brain-injured mice spent a remarkably similar amount of time engaged in social investigation of the intruding female, averaging a cumulative time of 241.7±13.7 and 247.6±20.2 sec, respectively (unpaired t-test, t_18_ = 0.24, p = 0.810). When analyzed by specific behaviors (figure 2a), a preference for sniffing the ano-genital region was again evident independent of injury (2-way RM ANOVA effect of behavior F_2, 36_ = 55.14, p<0.0001). Consistent with similar total investigative times, both sham and TBI mice spent comparable time participating in different social behaviors including sniffing head-torso, sniffing ano-genital, and following (no effect of injury F_1, 18_ = 0.06, p = 0.810). In contrast, mounting behavior was strikingly suppressed after brain injury (figure 1b). Compared to sham-operated mice, TBI mice showed a significant reduction in the number of mounting attempts towards the female stimulus (t_18_ = 2.85, p = 0.011). Ninety percent of sham-operated mice made at least one mounting attempt during the 5 minute test period, compared to only 70% of brain-injured mice. Further, latency from the initiation of the encounter until the first mounting attempt was delayed in TBI mice (t_18_ = 2.51, p = 0.021). Thus, TBI to the developing brain resulted in reduced sexual approach behavior despite normal social investigation towards a stimulus female.

Sociosexual investigation was also assayed at adulthood after TBI or sham-operation at adolescence (figure 2c). The total investigative time with the female stimulus was similar between sham and TBI mice, at 204.0±10.6 and 196.8±8.4 respectively (unpaired t-test, t_16_ = 0.53, p = 0.602). As with a male intruder, both sham and TBI mice spent the majority of this time engaged in ano-genital sniffing behavior, relative to head-torso sniffing and following (2-way RM ANOVA effect of behavior F_2, 32_ = 22.30, p<0.0001). In contrast to mice injured at p21, injury at p35 did not alter mounting behavior towards a novel female at adulthood (figure 2d). Instead, sham-operated and TBI mice showed a comparable number of mounting attempts (7.00±1.53 for sham, versus 5.89±1.36 for TBI; unpaired t-test t_16_ = 0.54, p = 0.594) and latency to the first mounting attempt (197.00±26.75 s for sham, versus 169.00±11.32 s for TBI; unpaired t-test t_16_ = 0.96, p = 0.349).

### Selective loss of social novelty preference after TBI at adolescence (p35)

**Figure 3:**
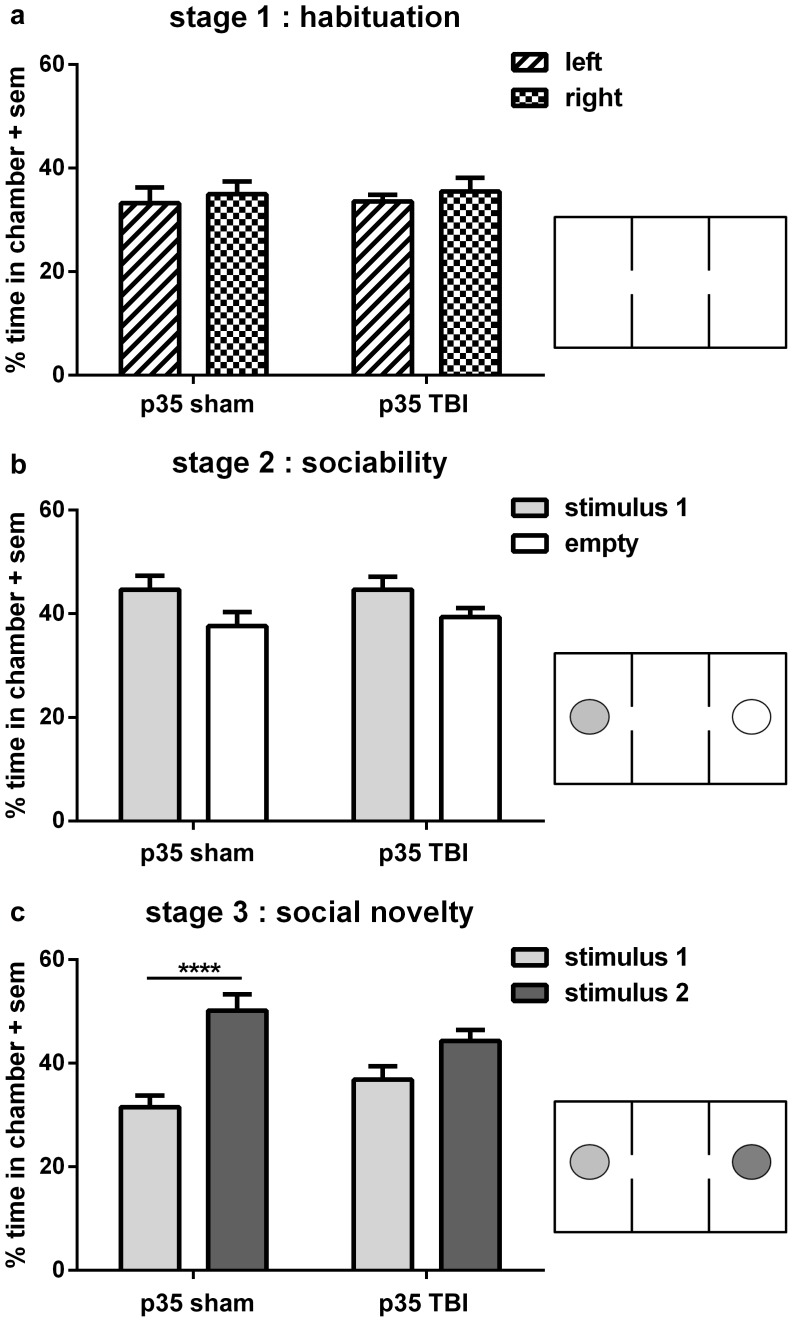
Loss of social novelty preference at adulthood is unique to TBI at p35. Independent of injury, all mice spent equal time in the left and right side chambers during habituation (stage 1; a). During stage 2 (b), both sham and TBI mice showed a similar preference for spending more time in the chamber containing the stimulus mouse, compared to the empty chamber (2-way ANOVA overall effect of stimulus, *p<0.05). In stage 3 (c), sham mice showed a strong preference for spending more time with stimulus mouse 2 compared to mouse 1, indicating social recognition or memory of ‘now-familiar’ stimulus 1 (2-way ANOVA post-hoc, ****p<0.0001). In contrast, TBI mice injured at p35 spent equivalent time with both stimulus mice (post-hoc, n.s.), indicating a lack of preference for social novelty.

We have previously identified deficits in sociability and social novelty recognition after TBI at p21 using the three-chamber social approach task [19]. Here, we also applied this paradigm to mice injured at adolescence (p35), to further elucidate age-dependent consequences to social deficits. As expected, both sham and TBI mice spent comparable time in each empty outer chamber during stage 1 habituation (figure 3a; 2-way RM ANOVA no effect of injury F_1, 32_ = 0.03, p = 0.868; no effect of chamber side, F_1, 32_ = 0.58, p = 0.452). Stage 2 (figure 3b) subsequently provided test mice with the choice between chambers containing either a novel stimulus male mouse or an empty cup. Here, both sham and TBI mice showed a similar preference for social proximity, spending more time in the chamber containing the stimulus mouse compared to the empty cup (2-way RM ANOVA overall effect of stimulus F_1, 32_ = 6.64, p = 0.015; no effect of injury F_1, 32_ = 0.130, p = 0.721; Bonferroni's post-hoc, n.s.).

Lastly, stage 3 challenged the test mice with a choice between proximity with the first stimulus mouse (now familiar) or a second novel stimulus male (figure 3c). Analysis by 2-way RM ANOVA revealed an overall effect of stimulus (F_1, 32_ = 26.16, p<0.0001) with no overall effect of injury (F_1, 32_ = 0.01, p = 0.914), however, interpretation of these main effects is confounded by a significant stimulus×injury interaction (F_1, 32_ = 4.75, p = 0.037). From Bonferroni's multiple-comparisons post-hoc analyses, it was evident that sham-operated mice showed a normal, strong preference for spending more time with the second stimulus mouse (p<0.0001), indicating intact social recognition. In contrast, the TBI group spent similar time in the chambers containing both the first and second stimulus mice (Bonferroni's post-hoc, p>0.05), indicating a lack of preference for social novelty.

### Reduced scent marking at adulthood after pediatric TBI

**Figure 4:**
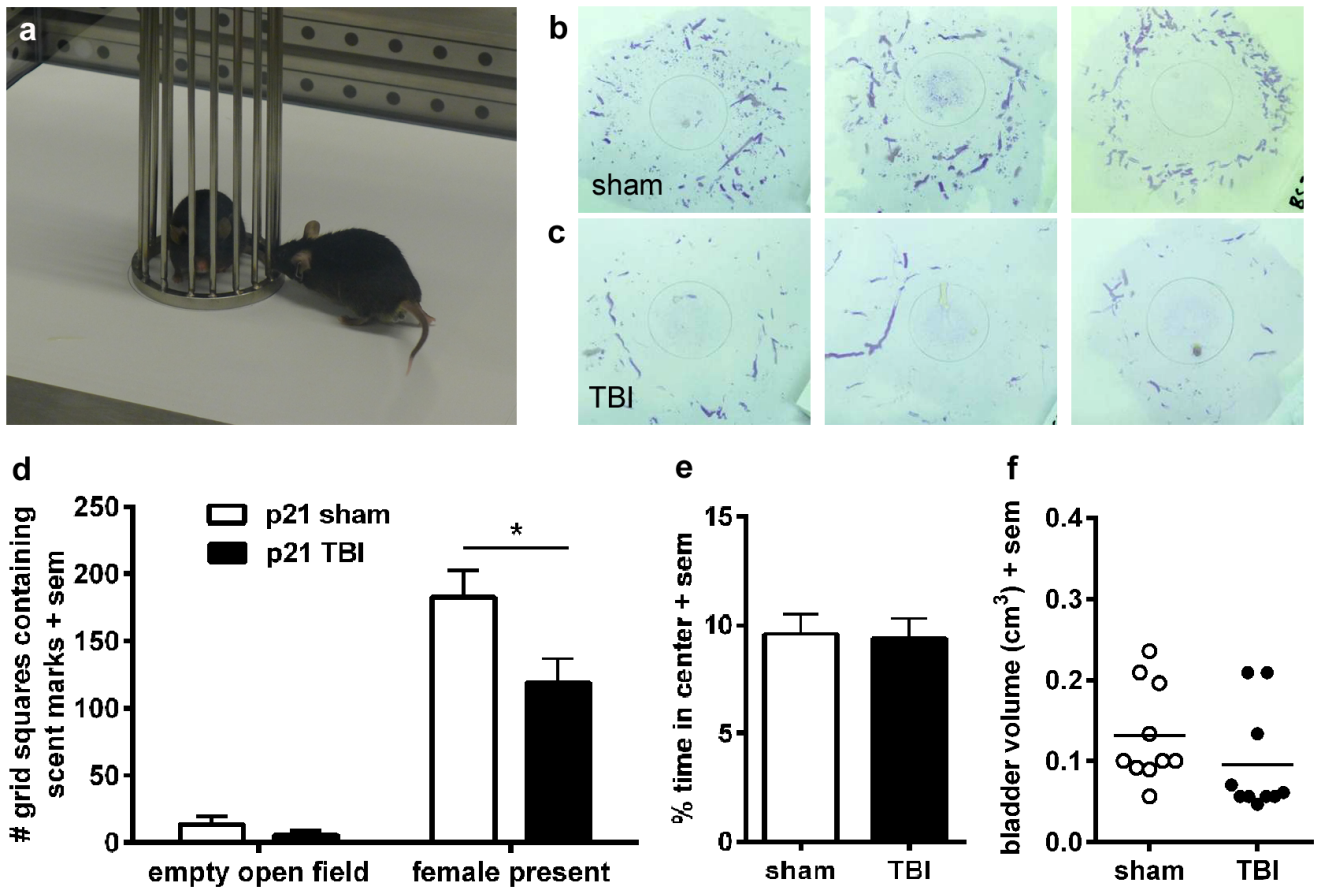
Reduced scent marking indicates impaired sociosexual communication after pediatric TBI. Scent marking by sham and TBI mice was assessed in an open field arena lined with absorbent paper, in the presence and absence of a cage-enclosed novel female mouse (a). Representative examples from sham mice and after injury at p21 are shown in panels (b) and (c), respectively. All mice produced more scent marks in the presence of a stimulus female compared to an empty arena (d). However, despite comparable production of scent marks at baseline (empty open field), TBI mice deposited significantly fewer scent marks in response to a novel female, compared to sham-operated mice (*p<0.05). This reduction was unlikely due to potential changes in mobility, anxiety or gross bladder dysfunction, as measures of anxiety (e) and bladder capacity (f) were comparable.

Scent marking in response to a novel female stimulus was assessed as an assay for sociosexual communication and recognition (figure 4a) [22]. In line with the use of this test in mouse models of autism, and based upon preliminary experiments, we restricted our analyses to the area surrounding the female stimulus as a better representation of communication and sociosexual motivation, as compared to the total number of scent marks deposited throughout the entire open field arena [26], [29]. After injury at p21, both sham and TBI mice deposited few scent marks in the arena in the absence of a stimulus female (figure 4d). Addition of a stimulus female to the arena dramatically stimulated scent marking behavior (2-way RM ANOVA effect of stimulus, F_1, 18_ = 100.40, p<0.0001), increasing the number of urine-containing grid squares 13-fold in sham-operated mice compared to when the arena was empty. Brain-injured mice also showed an increase in scent marking in the presence of a stimulus female compared to the empty arena; however, this response was ∼35% lower compared to sham-operated mice (2-way RM ANOVA effect of injury F_1, 18_ = 6.86 p = 0.017; stimulus×injury interaction F_1, 18_ = 3.93, p = 0.063; Bonferroni's post-hoc, p<0.05), indicating a reduced response towards a novel female mouse after injury (figure 4b, c). Defecation was also increased in the presence of the female stimulus compared to the empty arena (2-way RM ANOVA effect of stimulus F_1, 18_ = 7.03, p = 0.008). Defecation is thought to reflect emotionality in response to a stressful or novel environment [58], [59]. A non-significant trend towards reduced defecation was seen in brain-injured mice compared to sham (2-way RM ANOVA effect of injury F_1, 18_ = 3.99, p = 0.069).

There are several factors which may confound the interpretation of urine markings as social or sociosexual communication between mice – potential injury-related deficits in olfactory function, motor impairments, an anxiolytic phenotype, or problems with urination can influence scent marking behavior [26], [60]. However, we recently demonstrated that this injury model at p21 did not impair the ability to rapidly locate a buried odorous food source, providing evidence that olfactory function is intact in these mice [19]. Secondly, the total distance moved in the open field arena (in the absence of a female) was similar in sham-operated and TBI mice (5781±302.9 and 6195±374.9 cm, respectively; unpaired t-test, t_18_ = 0.86, p = 0.401), eliminating a deficit in mobility or general activity. Anxiety was also assayed in this context, by quantifying the time spent in the periphery versus the center of the open field arena (figure 4e). This measure was also similar between sham and TBI mice (unpaired t-test, t_18_ = 0.18, p = 0.857), such that reduced scent marking by brain-injured mice is unlikely to be a consequence of anxiety-related avoidance of the stimulus female.

In addition, scent marking was comparable between sham and TBI mice in the empty open field, suggestive of similar baseline urine output and normal bladder function in TBI mice (figure 4d). Lastly, gross bladder morphology was examined for potential injury-related hypertrophy or atrophy, by measurement of dissected weight and estimated volume [56]. Neither bladder weight (Mann-Whitney test, p = 0.164) nor estimated volume (Mann-Whitney test, p = 0.090; figure 4f) were significantly different between sham-operated and brain-injured mice, indicating the absence of gross bladder abnormalities. In summary, reduced scent marking towards a novel female mouse was evident at adulthood after TBI at p21, which cannot be attributed to olfactory, motor, anxiety or bladder-related deficits.

Scent marking towards a novel female mouse was also assayed after injury or sham-operation at p35 (figure 3). As expected, mice responded to the presence of a female mouse by scent marking the surrounding environment, quantified as an increase in the number of grid squares containing urine markings compared to in an empty arena (2-way RM ANOVA effect of stimulus F_1,16_ = 15.55, p = 0.001; Bonferroni's *post-hoc* p<0.05). However, this behavior was not altered by brain injury at adolescence (no effect of injury F_1, 16_ = 0.43, p = 0.523). Similarly, defecation was increased in the presence of a stimulus female compared to baseline (2-way RM ANOVA effect of stimulus F_1, 16_ = 9.22, p = 0.008), but this was similar in both sham-operated and TBI mice (no effect of injury F_1, 16_ = 0.01, p = 0.924).

### Emission of ultrasonic vocalizations after pediatric TBI is both stimulus and experience-dependent

Based upon the application of USVs as a potential measure of social communication in mouse models of communication disorders, we investigated whether USVs were altered after TBI at p21, the injury age after which we have seen the most robust social deficits emerge by adulthood. Two separate cohorts of mice were assessed; both received either TBI or sham-operation at p21 and were tested at ∼p80 in the same room under identical conditions of lighting, acclimatization and habituation. The key distinguishing feature between the cohorts was that Cohort 1 had previously undergone testing in the resident-intruder paradigm with both male and female stimulus mice approximately one week prior to USV recording, and thus had prior social experience (figure 1a). In contrast, Cohort 2 had no previous social encounters after being individually-housed (figure 1b). Mice that produced fewer than 5 calls during the 2 min recording period following addition of the stimulus were excluded as ‘non-responders.’ The number of non-responders which were excluded was roughly equivalent in sham versus TBI groups in both cohort 1 (n = 1/group for female stimulus; n = 0/group for female bedding; and n = 4/group for male stimulus) and cohort 2 (n = 0 TBI and n = 1 sham for female stimulus; n = 1 TBI and n = 2 sham for female bedding; and n = 2/group for male stimulus).

**Figure 5:**
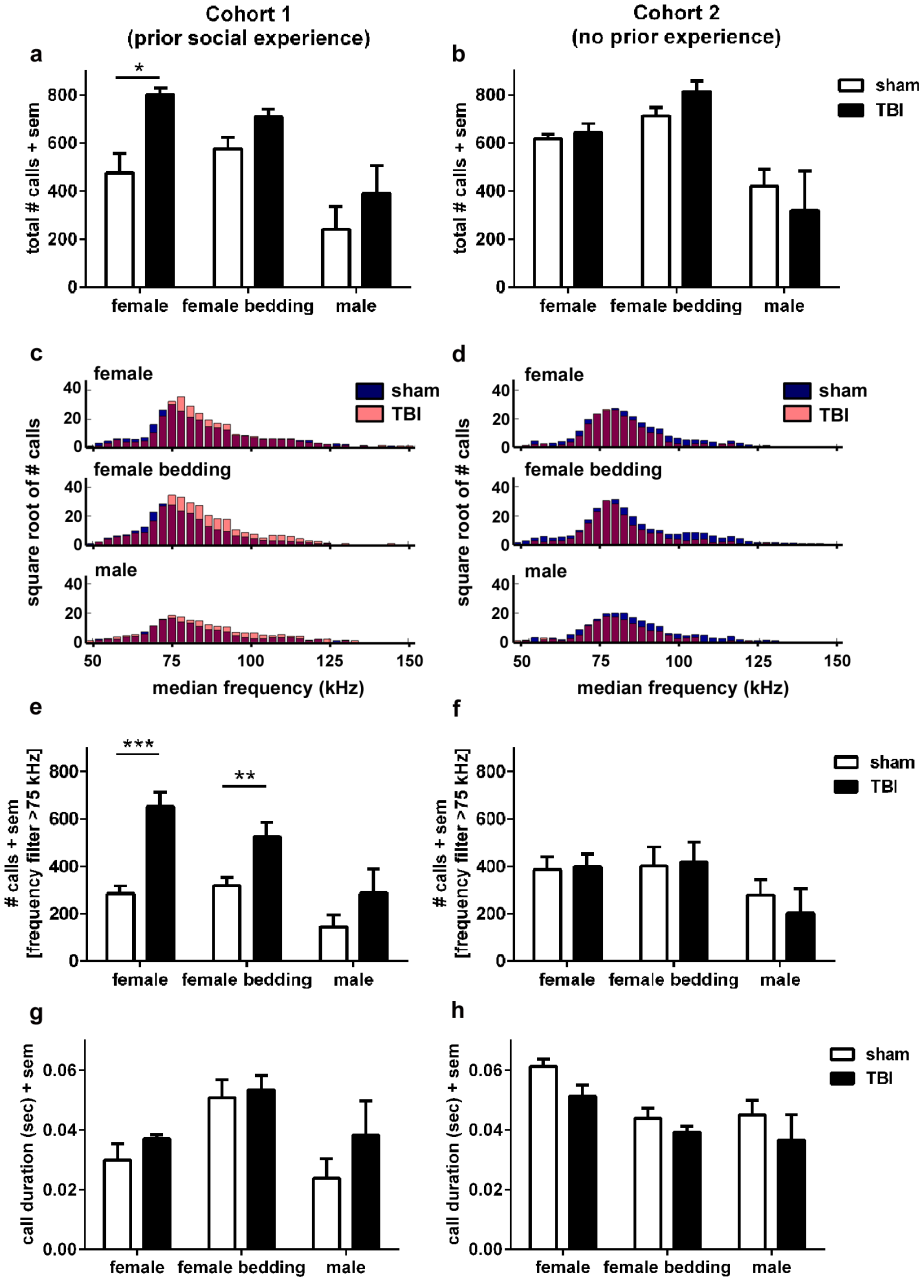
The number and duration of USV calls after injury at p21 are both stimulus and experience-dependent. USVs were recorded at adulthood after injury at p21, in response to three different stimuli; addition of a novel male mouse, novel female mouse, or bedding from a cage of novel female mice. In Cohort 1, which had previously encountered conspecifics of both genders during the resident-intruder test, TBI mice emitted significantly more total calls (a), particularly in response to a female stimulus mouse (*p<0.05). Histograms of the mean median call frequency revealed a distribution of calls above and below 75 kHz (c; dotted line). Application of a frequency filter to restrict analysis to calls greater than 75 kHz found that TBI mice produced more high frequency calls compared to sham mice in response to both a female mouse kHz found that TBI mice produced more high frequency calls compared to sham mice in response to both a female mouse (***p<0.001) and female bedding (**p<0.01). In contrast, a lack of prior social encounters as experienced by Cohort 2 (b, d, f) resulted in sham and TBI mice emitting a similar number of calls (both total and restricted to >75 kHz kHz). Call duration was not affected by injury in Cohort 1 (g), although in Cohort 2 (h) there was a trend towards TBI mice producing calls of shorter duration compared to sham mice overall (p = 0.069).

We first examined the total number of calls produced. For Cohort 1, sham-operated mice showed a high response to either a female mouse or female bedding, emitting an average of 476.17 and 575.83 calls respectively during the post-stimulus recording period (figure 5a). The number of calls was lower in response to a male stimulus mouse, averaging 240.17 total calls (2-way RM ANOVA effect of stimulus F_1, 9_ = 31.09, p<0.001). Brain-injured mice produced a greater total number of USV calls compared to sham mice (effect of injury F_1, 9_ = 6.15, p = 0.035). This difference was dependent upon the stimulus, with TBI showing a higher number of calls in response to a female mouse (F_1, 17_ = 8.23, p = 0.011) and a trend towards higher numbers of calls in response to female bedding (F_1, 19_ = 3.48, p = 0.078), but not in response to a male mouse (F_1, 11_ = 0.57, p = 0.468). The presentation of a male stimulus mouse elicited USVs from only a subset of mice (n = 6 sham and n = 5 TBI), and fewer calls from those that did vocalize. In contrast, presence of a female stimulus induced USVs in 90% of mice, and 100% of mice vocalized in response to female bedding.

The median frequency of emitted USVs was not affected by either stimulus (2-way RM ANOVA F_1, 8_ = 0.01, p = 0.848) nor injury (F_1, 8_ = 0.01, p = 0.911; Table S1). However, the generation of median frequency probability histograms revealed a consistent distribution around a peak at 75 kHz, with distinct populations of calls above and below this cutoff. Therefore a frequency filter was applied to examine the number of calls with median frequencies above 75 kHz [36]. Interestingly, TBI mice appeared to be producing a greater number of calls specifically in this higher range of frequencies (figure 5c). Quantification confirmed this observation (2-way RM ANOVA effect of injury F_1, 9_ = 136.74, p = 0.004), with TBI mice producing a higher number of calls compared to sham controls in response to both a female stimulus mouse (F_1, 17_ = 15.61, p = 0.001) and female bedding (F_1, 19_ = 11.39, p = 0.003; figure 5e), but not a male stimulus (F_1, 11_ = 0.93, p = 0.358). We next examined call duration (figure 5g), and found that this parameter was not affected by either TBI (2-way RM ANOVA effect of injury F_1, 9_ = 1.08, p = 0.325) or the stimulus type (F_1, 9_ = 0.19, p = 0.109). Lastly, the latency until the first vocalization after addition of the stimulus was similar in both TBI and sham-operated mice across all stimuli (Table S1).

In stark contrast, a different pattern of USVs was found when recording from mice with no prior social encounters (Cohort 2). The total number of USV calls (figure 5b) was again dependent upon the stimulus, as fewer calls were observed in response to males (2-way RM ANOVA effect of stimulus F_1, 8_ = 12.12, p = 0.008). However, TBI and sham-operated mice in this cohort produced a similar total number of calls regardless of stimulus (effect of injury F_1, 8_ = 0.02, p = 0.907). Consistent with this finding, there were also no differences in call number between TBI and sham mice when we applied the frequency threshold cutoff of 75 kHz (2-way RM ANOVA effect of injury F_1, 8_ = 0.03, p = 0.871; figure 5d, f). Mean call duration was also evaluated in Cohort 2 (figure 5h). In contrast to Cohort 1, TBI mice tended to exhibit shorter calls compared to sham-operated mice (2-way RM ANOVA F_1, 9_ = 4.259, p = 0.069). Specifically, a significant reduction in call duration was evident only in response to female mice (F_1, 9_ = 5.68, p = 0.044) but not in response to either female bedding or a male stimulus mouse (F_1, 9_ = 1.12, p = 0.321 and F_1, 9_ = 0.86, p = 0.381, respectively). As for all parameters, we re-analyzed these data with the additional of mice that did not respond to all three stimuli (total n = 6–8, 5–7 and 5–6 for female, female bedding and male stimuli, respectively), and therefore could not be included in the 2-way RM ANOVA analysis (n = 4–6/group). In this larger cohort, the response to female bedding approached significance (F_1, 12_ = 4.40, p = 0.060), with TBI mice again tending to produce shorter calls compared to sham animals. As for Cohort 1, the presentation of a male stimulus mouse elicited USVs from only a subset of Cohort 2 mice (n = 5–6 per group), and fewer calls from those that did vocalize. Neither call latency nor mean median frequency were altered by TBI in Cohort 2 (Table S1).

**Figure 6:**
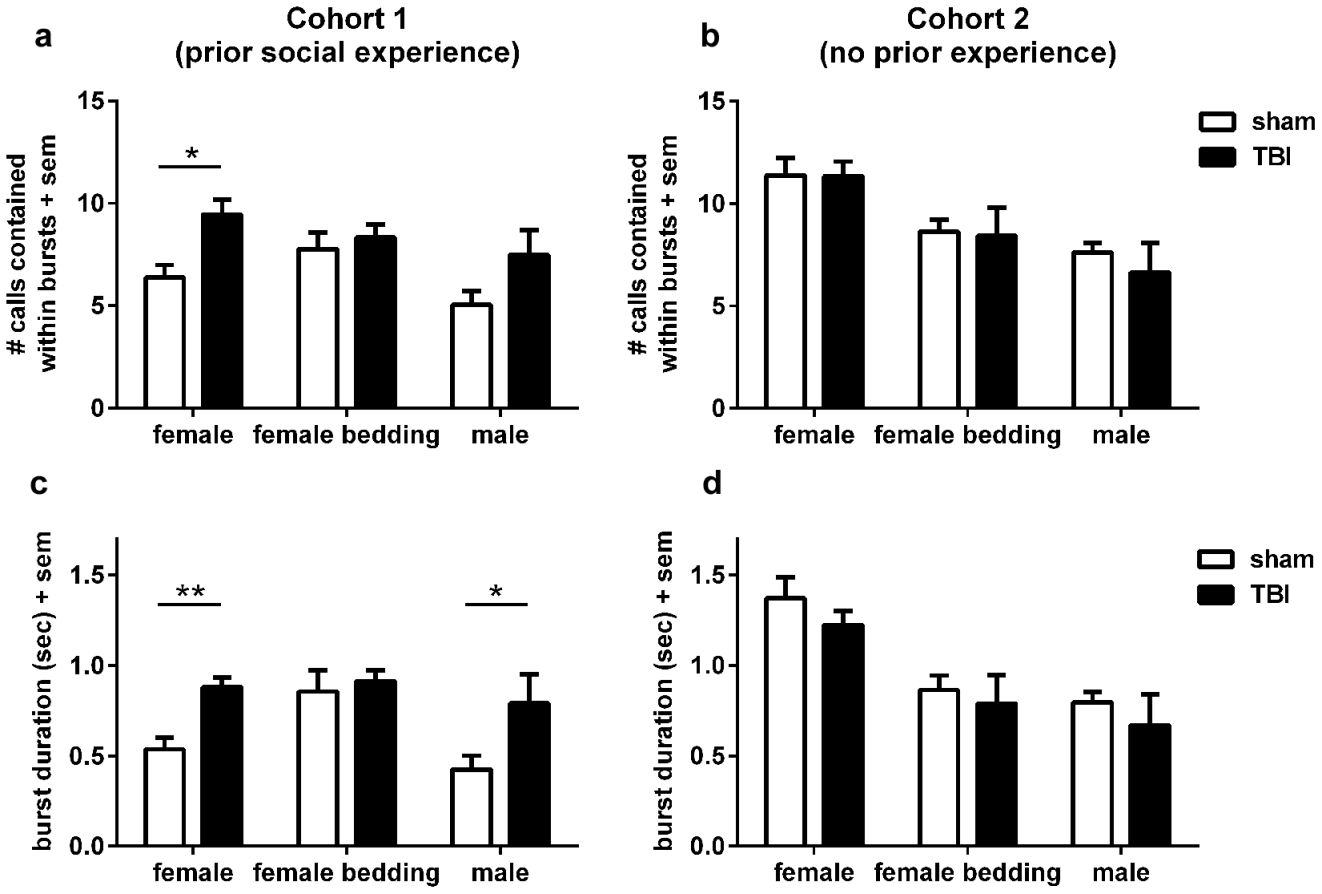
Burst analyses reveal temporal differences in USV calls after injury at p21. Call burst patterns were calculated from the distribution of inter-call intervals. In Cohort 1, the mean number of calls per burst was higher in mice after injury at p21 compared to sham mice in response to the female stimulus mouse (*p<0.05). Similarly, bursts of USV calls from TBI mice towards female stimuli were of a longer duration (c; **p<0.01), as well as towards a stimulus male (*p<0.05). In contrast, no such injury-related differences were observed in burst patterns produced in the absence of prior social experience (Cohort 2; b, d).

We next examined the burst patterns of USV calls to determine whether TBI altered the temporal organization of calls after addition of the stimulus. Call bursts were calculated from the distribution of inter-call intervals (ICIs). The fraction of calls contained within bursts was not affected by TBI (2-way RM ANOVA effect of injury, F_1, 10_ = 3.04, p = 0.109), averaging 0.91 (or 91% of total calls) across both groups and all stimuli (Table S1). We next assessed the mean number of calls per burst in Cohort 1 (figure 6a), and found a trend towards an effect of injury (2-way RM ANOVA F_1, 8_ = 5.244, p = 0.051). This was most evident in response to a female stimulus, where TBI mice produced an average of 9.45±0.72 calls within a burst compared to 6.37±0.62 for sham-operated mice (one-way ANOVA F_1, 9_ = 10.12, p = 0.013). This difference corresponded to a similar distinction in the mean burst duration in Cohort 1 (figure 6c), whereby TBI mice produced USV bursts of longer duration compared to sham mice in response to a female stimulus (2-way RM ANOVA effect of injury F_1, 8_ = 5.19, p = 0.052; one-way ANOVA F_1, 9_ = 13.32, p = 0.006). No trends or statistically significant differences were found in the burst parameters for female bedding or male mouse stimuli.

Again, burst analysis of USV calls produced by Cohort 2 was conflicting (figure 6b, d). Here, we found no differences or trends between TBI and sham mice in terms of the fraction of calls within bursts (Table S1; 2-way RM ANOVA F_1, 10_ = 3.04, p = 0.112), mean burst duration (F_1, 10_ = 0.96, p = 0.350), or mean number of calls within bursts (F_1, 10_ = 0.17, p = 0.687).

### White matter atrophy at adulthood after pediatric and adolescent TBI

**Figure 7:**
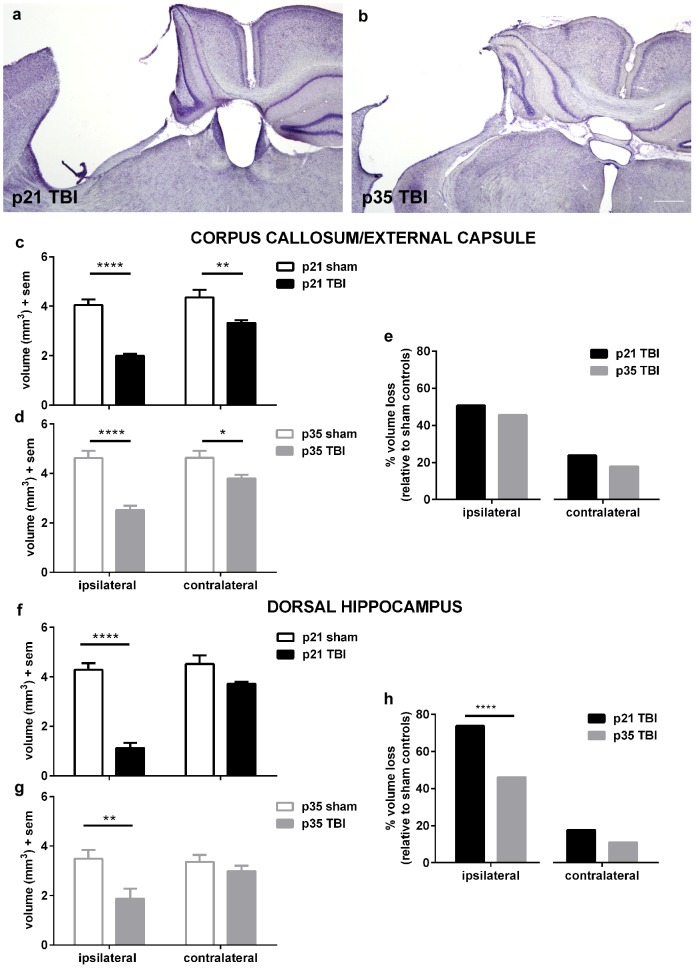
Social deficits are associated with white matter atrophy and hippocampal loss. TBI at both a pediatric (a) and adolescent age (b) resulted in a distinct pattern of unilateral cortical and hippocampal loss by adulthood (scale bar = 500 µm). Volumetric analysis of the corpus callosum revealed pronounced volumetric loss of the corpus callosum/external capsule bilaterally after injury at p21 (c; ****p<0.0001, **p<0.01). Similar loss was also evident after injury at adolescence (d; ****p<0.0001, *p<0.05). When expressed as injury-induced tissue loss relative to age-matched sham controls after injury at p21 compared to p35 (e), both cohorts showed ∼50% volume loss of the ipsilateral corpus callosum and ∼20% loss on the side contralateral to injury (n.s.). Volume of the dorsal hippocampus was also quantified, revealing considerable loss of the ipsilateral hippocampus after injury at p21 (f) and p35 (g). When expressed as tissue loss relative to age-matched sham controls (h), greater tissue ipsilateral hippocampus loss was evident after injury at p21 compared to injury at adolescence.

As social dysfunction after TBI in patients has been associated with white matter damage, we examined volumetric changes in the corpus callosum/external capsule as a measure of long-term white matter integrity (figure 7). At adulthood after injury at p21 (figure 7c), an overall reduction in the volume of the corpus callosum was evident (2-way RM ANOVA effect of injury F_1, 8_ = 29.75, p<0.0001), which was dependent upon proximity to the impact site (effect of region F_1, 8_ = 219.10, p = 0.0006; injury×region interaction F_1, 8_ = 85.76, p<0.0001). Of note, atrophy of the corpus callosum was observed both ipsilateral to the impact site (Sidak's post-hoc, p<0.0001), as well as contralaterally (p<0.01).

After TBI at adolescence (figure 7d), we also found a distinct loss of corpus callosal volume by adulthood (2-way RM ANOVA effect of injury F_1, 13_ = 24.04, p = 0.0003), which was again dependent upon the hemisphere (effect of region F_1, 13_ = 130.90, p<0.001; injury×region interaction F_1, 13_ = 125.00, p<0.0001). Atrophy was greatest in the corpus callosum ipsilateral to the injury (Sidak's post-hoc, p<0.0001) but also detected contralaterally (p<0.05).

To directly compare volumetric loss after injury at p21 relative to p35, we calculated the percentage loss for each TBI group relative to their age-matched sham controls, as we hypothesized that the extent of white matter damage after injury at a younger age may contribute to the greater extent of social deficits seen after injury at p21 (figure 7e).Contrary to our hypothesis, however, the corpus callosum showed comparable tissue loss between cohorts. The ipsilateral corpus callosum after injury at p21 was reduced by 50.7%, compared to after p35 injury when 45.8% of the ipsilateral corpus callosum was atrophied (Fisher's exact test, p = 0.5715). Similarly, after injury at p21 the contralateral corpus callosum was reduced by 23.8%, compared to 17.9% after injury at p35 (Fisher's exact test, p = 0.3856).

### Greater hippocampal loss at adulthood after pediatric TBI

We lastly evaluated changes to the injured dorsal hippocampus, as this region is postulated to be involved in social memory and recognition functions [61], [62], and is known to be affected in this model [38]. After injury at p21 (figure 7f), considerable hippocampal loss was evident (2-way RM ANOVA effect of injury F_1,8_ = 34.26, p = 0.0004; effect of region F_1, 8_ = 267.60, p<0.0001; interaction F_1, 8_ = 187.40, p<0.0001). In contrast to the more diffuse, bilateral nature of white matter atrophy, tissue loss was confined to the hippocampus ipsilateral to the injury (Sidak's post-hoc, p<0.0001). A similar, region-specific response was seen after injury at p35 (figure 7g), whereby hippocampal volume was reduced ipsilateral to the impact site (Sidak's post-hoc p<0.01; 2-way ANOVA effect of injury F_1, 13_5.79, p = 0.0317; effect of region F_1, 13_ = 4.02, p = 0.0664; interaction F_1, 13_6.38, p = 0.0253).

To directly compare changes in the dorsal hippocampus after injury at p21 relative to p35, we next calculated the percentage loss for each TBI group expressed relative to their age-matched sham controls (figure 7h). By this method, we saw significantly greater loss of the ipsilateral hippocampus after injury at p21 (73.7% loss) compared to injury at p35 (46.17% loss; Fisher's exact test, p<0.0001). In contrast, loss of contralateral hippocampus was comparable across both age groups (p = 0.2278).

## Discussion

Like humans, mice are inherently social beings that exhibit a rich repertoire of social abilities and complex interactions which mature during childhood and adolescence [63]. Here, we utilized this similarity to examine potential changes in social interactions and social communication as a result of TBI. We focused on outcomes at adulthood after pediatric and adolescence injury, based upon clinical evidence that social impairments persist long-term well into adult life [11], [12], [64], [65], as well as previous studies in our laboratory indicating that social deficits develop over time after injury to the developing mouse brain [19]. A key finding from the present study was that brain injury at p21 resulted in reduced scent marking and mounting behavior towards an unfamiliar female at adulthood, in addition to reduced social investigation towards an unfamiliar male. These results suggest that injury at an early age may disrupt the normal development of social behaviors, leading to the emergence of social and sociosexual deficits at adulthood. In contrast, mice injured at adolescence (p35) showed remarkable resilience to such impairments, suggesting that vulnerability of the immature brain to psychosocial dysfunction is maturation-dependent.

### Scent marking behavior – a new assay for TBI-induced communication impairments

Male mice actively deposit urinary scent marks towards adult females, their urine, saliva and vaginal fluids [25]. The assessment of scent marking behavior is increasingly utilized in mouse models of autism spectrum disorders and language impairments, and represents a useful, ethologically-valid measure of social communication in mice [22], [24], [66]. Of note, scent marking has recently been proposed as a measure of hedonic or reward-seeking behavior, which is sensitive to psychosocial stressors [67]. Given the similarities between social impairments seen in individuals with autism and TBI, we hypothesized that this test might also detect non-verbal communication deficits after experimental TBI. Indeed, we saw a striking reduction in scent marking behavior by adult mice after TBI at p21. Factors which might influence scent marking including general activity, anxiety, bladder capacity and olfactory function, were not impaired by TBI, nor was baseline deposition of urine under non-social conditions (the empty open field arena) altered in TBI mice. Therefore, we interpret the reduced scent marking behavior in TBI mice in a sociosexual context to likely reflect a specific deficit in social behavior or sexual motivation.

To our knowledge, this is the first application of a urine scent marking task in the context of experimental TBI. The task has both construct validity (a similar underlying cause of disease/injury) and face validity (resemblance to human symptoms). The testing paradigm is fairly straight-forward and can be adapted to the use of existing open field arenas, and future automation of quantification using imaging software will further accelerate data analysis from this assay. We propose that incorporating this paradigm across different TBI models, in concert with measures of social investigation, may provide important insight into psychosocial outcomes after TBI in relation to different injury severities and mechanisms.

### Reduced sociosexual behaviors after early life TBI

Clinical studies provide evidence of abnormal sexual behavior in a substantial proportion of patients after TBI, apparent as reduced sexual drive and arousal that is not solely associated with physical injury or mobility problems [68], [69], although an increased prevalence of inappropriate or offensive sexual behaviors has also been reported [70], [71]. In the current study, we found a reduction in mounting behavior towards a novel female mouse after TBI at p21, which may reflect sexual anhedonia at this time. Interestingly, this reduced mounting behavior was evident despite a similar amount of time spent in general social interactions with the female stimulus, suggesting that a reduction in mounting attempts is not simply a reflection of increased social anxiety. Our findings are consistent with Pandey and colleagues, who demonstrated impairments in social and sociosexual interactions after a weight-drop injury in adult rats [72]. The authors interpreted these changes as ‘depressive-like behaviors,’ based upon the understanding that a loss of sexual interest (sexual anhedonia) is one of the cardinal symptoms of depression [73]. Although we did not measure depressive behaviors *per se* in the current study, this would be worth pursing in the future, particularly in relation to clinical evidence that psychosocial stressors contribute to depressive symptoms and psychological distress after TBI [74], as well as an emerging association between depression and compromised sexuality amongst TBI survivors [75], [76]. An alternative possibility is that sexual anhedonia may reflect injury-induced disturbances in the endocrine system and reduced levels of key neuropeptides and androgens [67], [77], [78]. Recent clinical studies have highlighted the prevalence of endocrine disturbances after TBI in both children and adults [79], [80], [81], whereby pituitary function may appear normal acutely but become impaired over months post-injury [82], [83].

### Age-dependent vulnerability to social deficits

Clinical evidence supports the hypothesis that a younger age-at-insult results in increased vulnerability to social and communication deficits [6], [9], [84], and that a younger age at injury is more likely associated with global impairments and a lower quality of life [85]. For example, the older children are when they sustain a TBI, the fewer restrictions they experience in terms of social participation [6]. Underlying this idea is our understanding that the child and adolescent brains are still undergoing considerable development, and that injury disrupts both current social abilities as well as the subsequent trajectory [15]. Further, the acquisition of new social skills may be impaired by concurrent cognitive and neurological deficits which are typically associated with TBI at a young age [86], [87].

Here, we explored this hypothesis by comparing mice that received TBI at either p21 or p35, representing toddler and adolescent age groups, respectively [18]. After weaning (∼p21) is the time when social play behaviors typically become more prevalent, peaking around p25–40 [18], [46]. The same injury parameters were utilized for both groups of mice, based upon similarities in terms of gross brain size at these ages [88], [89]. Compared to reduced social investigation of a novel stimulus mouse after injury at p21, mice injured during adolescence exhibited normal sociability towards both male and female stimulus mice, as well as normal sociability in the three-chamber social approach task (stage 2). The three-chamber task was performed previously by our laboratory in mice injured at p21 [19], and is a useful supplementary assay of social behavior specifically limited to social approach by the test mouse, rather than reciprocal social interactions as in the resident-intruder paradigm. Here, mice injured at adolescence showed a subtle, selective loss of a preference for social novelty, spending equivalent time with a familiar mouse compared to a novel stimulus mouse during stage 3 of the task. While we cannot exclude the possibility that general memory impairment may underlie this observation [62], the finding is suggestive of a deficit in social recognition and/or social memory in this context [49], [63], [90]. The ability to recognize others is a fundamental component of social interaction, and adolescence in particular is a time characterized by continuing improvement in facial recognition and emotionality in humans [91]. Although not explored in the current study, differentiating between general memory dysfunction from a specific deficit in social recognition requires further investigation, for example, by application of novel object recognition or spatial water maze tasks.

### Ultrasonic vocalizations

Male-emitted USV's have been described as an index of social and sexual interest, recognition and motivation [54], [66], although the precise relationship of these calls to human communication remains the focus of intense debate [92], [93]. Based upon changes in USVs reported in several genetic models of autism, which may reflect a similar spectrum of social deficits to that seen after pediatric TBI [26], [29], [35], [36], [37], we hypothesized that injury to the developing brain would also result in altered vocalizations in a social or sociosexual context. Only one other study to date has considered USVs in the context of a mechanical brain injury - Matsumoto and colleagues demonstrated that direct bilateral lesions of the amygdala in adult male mice reduced syllable duration and long syllable frequency, which corresponded to a reduction in mounting behavior in the same mice [94].

Consistent with previous reports that female body and urinary odors alone are sufficient to trigger USVs from male mice [30], [31]. Here we saw a robust vocalization response to female bedding as well as to a female stimulus mouse. One major caveat of recording from male-female encounters is the potential for confounding vocal responses by the female mouse, as well as the possibility that she may respond differently to USVs emitted by brain-injured or sham mice to either encourage or inhibit ongoing calls. The paradigm of female bedding as the stimulus avoided this potential confound, and also differs in that it was collected from a communal cage of 4–5 stimulus females and thus likely presents additional olfactory cues. Interestingly, we typically saw consistent results across these two stimuli, suggesting that female bedding alone may provide a useful paradigm for investigating vocalizations in a sociosexual context. We also saw a stimulus-dependent response in all mice, whereby they vocalized more in response to female stimuli compared to males [95]. While some authors have reported that USVs are not detectable during male-male agonistic encounters [66], [96], [97], [98], we found that a subset of both sham and TBI mice did indeed vocalize towards a stimulus male, consistent with recent data using a similar recording paradigm [35].

Our key finding here was that TBI mice that had previously encountered a female mouse (Cohort 1) emitted an increased total number of USVs compared to sham controls in response to female bedding or an unfamiliar female stimulus. Specifically, TBI mice produced more high frequency calls (>75 kHz). Further, TBI mice produced a greater number of calls within bursts, which were typically longer in duration compared to bursts of calls from sham mice. This difference was not seen in response to the male stimulus, highlighting the relevance of the social context in which mouse communication is evaluated. Several previous studies have suggested a correlation between USV production and courtship behaviors [32], [99], [100]. However, here we paradoxically saw an *increased* number of calls made by TBI mice, which are also characterized by a reduction in other sociosexual behaviors such as scent marking and mounting. Social status is thought to play an important role in vocalizations between rodents, and dominant male mice have been shown to emit more USVs at ∼70 kHz in response to females compared to subordinate males [95]. This is worth noting in the context of our recent finding that brain-injured mice show an increased tendency for social dominance at adulthood [19]. Future studies may incorporate simultaneous and synchronized video-monitoring of investigative behaviors with USV recordings to delineate the temporal relationship between calls and particular behavioral phenotypes.

This striking finding of increased USV production after TBI was not replicated in mice that had no prior experience with females (Cohort 2), suggesting that prior social encounters ‘primes’ mice to emit USVs in an experience-dependent manner. There is conflicting data in this regard, mostly in terms of whether male mice will vocalize in response to female urine in the absence of previous female experience [23], [101], [102], [103]. It has been proposed that adult heterosexual encounters will elicit a greater USV response due to associative learning or classic conditioning [102], and others have demonstrated that a 5 min encounter with an adult female (as experienced by Cohort 1) is sufficient experience to promote subsequent vocalizations [23], [26]. However, our data suggest a greater level of complexity; sham mice, which had not previously encountered adult females prior to USV recordings (Cohort 2), in fact produced a similar number of total calls comparable to sham mice in Cohort 1, albeit with a delayed onset (Table S1), consistent with other reports of high levels of USV emissions in sexually naïve mice [104]. Here, we found that prior social experience, a more biologically-relevant paradigm compared to a lack of previous encounters, appeared to alter the vocal response specifically of brain-injured mice. We surmise that USV production after brain injury is both context and experience-dependent, and requires further investigation.

### Anatomical correlates

Social behaviors in humans are mediated by the ‘social brain,’ a complex network of interconnected neuroanatomical regions including the superior temporal sulcus, fusiform gyrus, temporal pole, medial prefrontal cortex, orbitofrontal cortex, hippocampus, amygdala, temporoparietal junction, and inferior parietal cortex [91], [105]. By conventional and functional magnetic resonance imaging, these regions show changes in structural reorganization, connectivity and activity during performance of socio-cognitive tasks at childhood and adolescence, indicating considerable ongoing development during brain maturation [106]. After TBI, associations between social deficits and frontal neuropathology have been reported clinically [107], [108], [109], and focal lesions to specific brain regions implicated in social cognition and information processing can manifest as social dysfunction [109]. However, in our injury model, a focal controlled cortical impact was focused over the parietal lobe with minimal if any structural damage to the frontal lobes. Further, we recently demonstrated that a unilateral lesion specifically to the frontal lobe of mice at p21 fails to elicit social deficits [110]. Together, these findings support a more global mechanism underlying abnormal social function after TBI.

Indeed, there is now increasing evidence that diffuse axonal injury, which disrupts white matter connectivity between regions of the social brain network, may underlie many TBI-induced social deficits [64]. Atrophy of the corpus callosum was evident 7–10 years after pediatric TBI in two independent patient cohorts [111], [112], and involved both volumetric loss as well as evidence of microstructural damage [113]. Further, greater loss of corpus callosal volume has been correlated with poorer social outcomes [114] and poorer emotion perception [64].

Here, we focused on the corpus callosum as the largest white matter tract in the mouse brain, which appears to be both directly and indirectly affected by experimental TBI [115]. Based upon this literature, we hypothesized that global loss of white matter would be more pronounced after injury at p21, in line with greater social deficits seen in this cohort compared to injury at adolescence and the presumption that the extent of white matter atrophy contributes to poorer social outcomes. On the contrary, we in fact found comparable bilateral atrophy of the corpus callosum/external capsule after both pediatric and adolescent injury. It is worth noting that several other studies have failed to establish an association between corpus callosum volume or midline thickness and sociability in mice, when comparing different mouse strains or examining the consequences of early-life callosal transection [41], [42], [116], [117]. However, the volumetric analysis conducted here, while in line with analyses in several clinical studies, does not exclude potential differences in regional microstructural integrity which may influence white matter connectivity. Volumetric changes likely results from the retardation of normal growth in addition to ongoing neurodegeneration [118], and there is also the potential for aberrant growth or hypertrophy to occur, particularly in the setting of ongoing brain development. Techniques such as diffusion tensor imaging may be required to elucidate the complex relationship between neural circuitry, connectivity of specific pathways and social behaviors.

We also examined volumetric changes in the hippocampus, a region postulated to be involved in social learning and memory functions, and directly impacted by injury in this model. Here, we found that loss of the ipsilateral hippocampus was significantly greater after injury at p21 compared to p35. As the injury parameters were equivalent at both ages, these data suggest that lesion progression may be more advanced by the time of tissue collection after p21 compared to after injury at p35. The implication of this finding is that reduced social deficits seen after injury at p35 may in fact be attributed to assessment at a time when lesion expansion was not yet completed. An alternative explanation is that the younger brain may show greater vulnerability to ongoing pathogenesis. Similar atrophy in the corpus callosum, together with differential volumetric loss of the hippocampus in the two cohorts, suggests that the kinetics of ongoing pathogenesis may be dependent on both the brain region and developmental age. Further research is required to validate one of these interpretations.

### Clinical implications

Although persistent aphasia is relatively uncommon following brain injury, deficits in communication skills are widely reported both in terms of the ability to produce effective language and understand language in context [119]. Language and communication problems are likely to negatively impact social abilities in brain-injured individuals, with reports that TBI survivors frequently experience difficulty with non-literal comprehension, discourse processes and communicating their thoughts [6], [7]. Impairments in recognizing and interpreting non-verbal emotional cues are common in children after TBI [120], [121], as well as pragmatic communication deficits including understanding sarcasm, differentiating truth from deception, using complex language, and comprehending figurative language such as metaphors [122]. Communication impairments may also manifest as excessive talkativeness, tangential and inappropriate conversation [123], [124], [125]. The consequences of such deficits are negative long-term outcomes including rejection by peers and limitations related to education and employment [2], [122]. By identifying social and sociosexual dysfunction in mice after experimental TBI, we here demonstrate the relevance of this animal model for future studies to investigate underlying mechanisms as well as target these clinically-important symptoms with novel therapeutics.

Our findings also have implications for rehabilitation strategies. Evidence of rehabilitation for social cognition following brain injury is sparse, and existing social skill treatment programs have shown limited efficacy [124]. Given the protracted nature of psychosocial deficits after TBI at a young age, and the delayed onset of these abnormalities, our work aligns with clinical evidence highlighting the need for both acute interventions and very long-term follow-up targeting social skills in TBI survivors [10].

### Limitations and future directions

There are a few caveats to keep in mind. In particular, the estrous state of the female stimulus mice was not monitored in this study. Previous research has shown that male mice deposit scent marks at high levels in response to females at all points in the estrous cycle, suggesting that the estrous state should not affect the amount of scent marking in males [24], [126]. In line with this, male mice have been shown to mount females of all estrous states equally [32]. However, we cannot rule out the possibility that the sexual responsiveness of the female mouse may have influenced the test male's behavior in some way. Further, these is some evidence that pheromone signaling from females may influence USV production by male mice [32], [127]. As such, we recommend that estrous state of stimulus female mice should be controlled for in future experiments.

Another caveat is the time post-injury when behaviors were assessed. We evaluated behavior at adulthood after injury at p21 or p35, based upon previous findings of an age-dependent emergence of functional deficits after pediatric TBI [19]. Both cohorts were evaluated at the same developmental age, based upon evidence that social behaviors are age-dependent up to at least p90 [41], [42], [43], [44]. However, this design required that the post-injury time period was approximately two weeks longer after injury at p21 compared to mice which received injury at p35. Considering clinical evidence suggesting that impairments may take several years to manifest [128], it is possible that the lack of deficits seen after injury at adolescence may reflect the shorter time interval between injury and assessment compared to mice injured at p21. Supporting this hypothesis, and as mentioned above, we found a greater extent of hippocampal loss after injury at p21, suggesting that long-term neurodegenerative processes may not yet have stabilized to the same degree in both cohorts [129], [130]. Further complicating the age comparison, we cannot rule out the possibility that the rate of lesion progression may be unique to injury at different ages, potentially due to age-dependent differences in secondary injury processes [131], [132].

Lastly, it is also worth reflecting that we are only just beginning to understand how social interactions in rodents relate to the complexity of human behaviors. Future studies may incorporate additional tests to further elucidate the utility of rodent models in the context of experimental brain injury, for example, with paradigms of social habituation and discrimination, testing for memory of a previously-established social hierarchy [133], and tasks to evaluate social information processing and social learning [134].

## Conclusions

In this study we have identified social and sociosexual dysfunction in mice at adulthood after pediatric TBI, which parallel some of the phenotypes observed after TBI in young patients. In addition to reduced social investigation towards conspecific males, brain-injured mice showed reductions in sexual mounting behavior and scent marking, as well as an increase in the number of high frequency USVs in biologically-relevant sociosexual contexts. Further, the manifestation of social dysfunction after injury at a younger age was associated with greater relative loss of the dorsal hippocampus, whereas volumetric changes in white matter were pronounced independent of age-at-injury, suggesting that the kinetics of secondary pathogenesis underlying these behavioral changes may be region and age-specific. These data lend support to the hypothesis that the immature brain shows vulnerability to early life disruption [64]. In line with this, it has been proposed that sociocognitive impairment after childhood TBI may reflect a failure to develop and acquire skills at an age-appropriate rate [15], [17]. Social and communication-related disabilities after childhood TBI may be secondary to problems in executive function, cognition, memory and behavioral impairments [84], and indeed, the social deficits seen in our p21 injury model emerge over time post-injury [19] and coincide with the appearance of cognitive deficits [39]. Future studies tracking post-traumatic neurodegeneration across development are needed to determine whether subtle abnormalities in white matter or hippocampal connectivity underlie these social changes.

## Supporting Information

Figure S1 **Body weights post-surgery.** Body weights were monitored at 1, 3 and 7 days post-surgery then weekly thereafter. There were no differences between TBI and sham-operated mice across time (2-way RM ANOVA with factors of injury and time, n.s.), demonstrating good general health of both injured and sham-operated mice.
(TIF)

Figure S2 **Apparatus for USV recording.** Enclosures used for testing with female bedding (a) or the addition of a male or female stimulus mouse (b). An Avisoft UltraSoundGate CM16/CMPA microphone was suspended above the enclosure, which was placed either on the bench top (Cohort 1) or within a sound-attenuating box (Cohort 2).
(TIF)

Figure S3 **Scent marking at adulthood is unaffected by brain injury at adolescence.** Urinary scent marks was similarly elevated by sham and TBI mice in response to a female stimulus compared to the empty open field (a). The ability to deposit scent marks was not affected by injury-related changes in anxiety or bladder structure, as sham and TBI mice spent a similar amount of time in the center of the open field (b) and exhibited similar bladder volumes upon autopsy (c).
(TIF)

Table S1 **Call parameters not affected by injury in either cohort.**
(DOCX)
