# Supplementary figures and images for: Sociosexual and Communication Deficits after Traumatic Injury to the Developing Murine Brain
Source: PLoS One. 2014 Aug 8;9(8):e103386. doi: 10.1371/journal.pone.0103386 (PMC4126664; doi:10.1371/journal.pone.0103386)

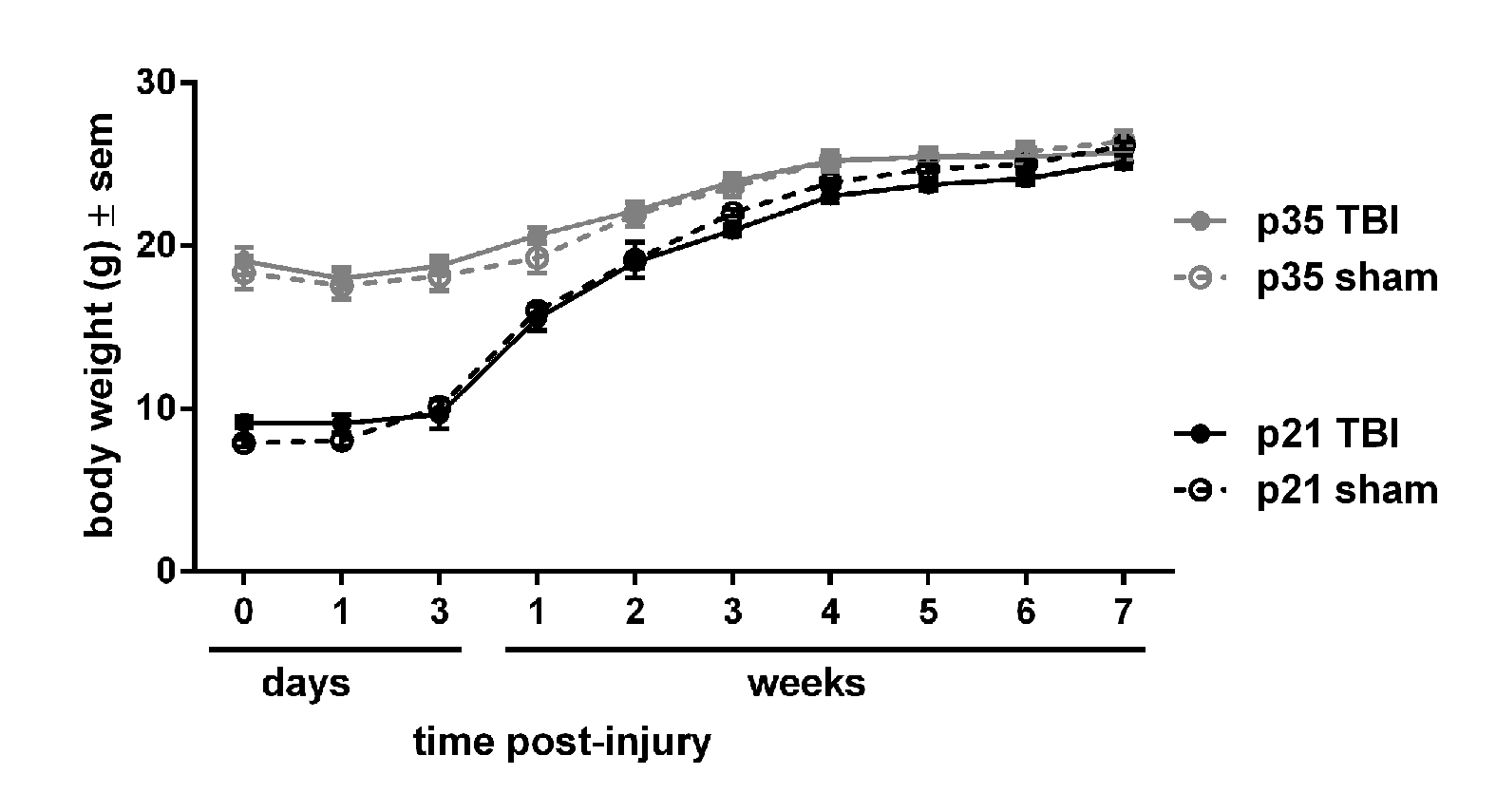

Supplement: Figure S1 — Body weights post-surgery. Body weights were monitored at 1, 3 and 7 days post-surgery then weekly thereafter. There were no differences between TBI and sham-operated mice across time (2-way RM ANOVA with factors of injury and time, n.s.), demonstrating good general health of both injured and sham-operated mice. (TIF) [file pone.0103386.s001.tif]

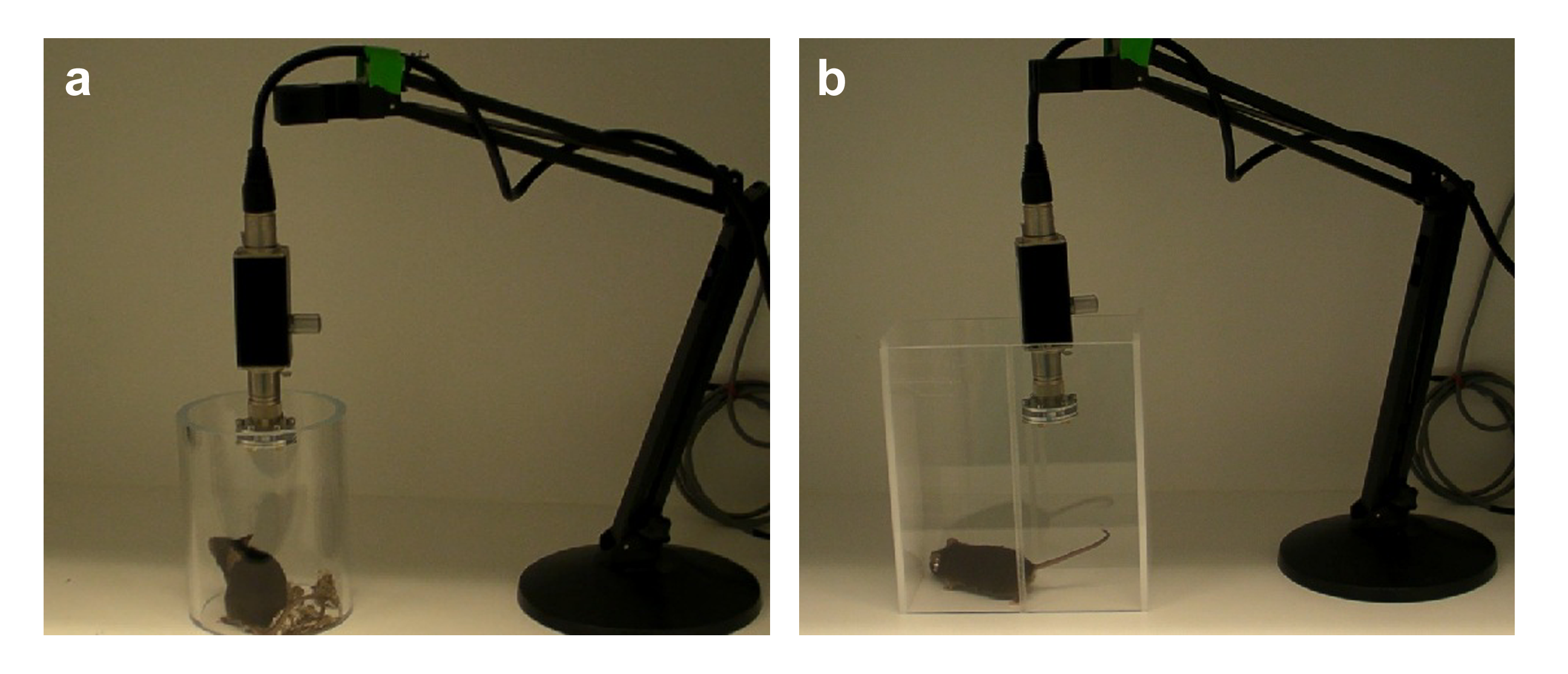

Supplement: Figure S2 — Apparatus for USV recording. Enclosures used for testing with female bedding (a) or the addition of a male or female stimulus mouse (b). An Avisoft UltraSoundGate CM16/CMPA microphone was suspended above the enclosure, which was placed either on the bench top (Cohort 1) or within a sound-attenuating box (Cohort 2). (TIF) [file pone.0103386.s002.tif]

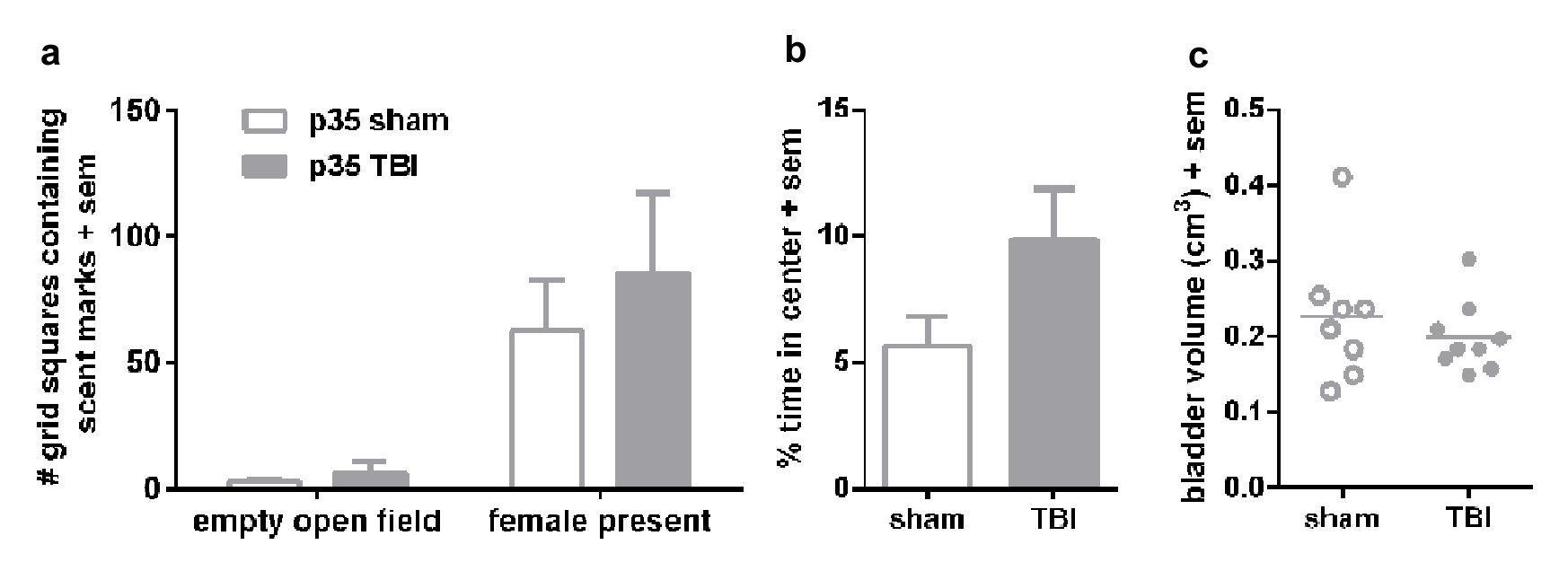

Supplement: Figure S3 — Scent marking at adulthood is unaffected by brain injury at adolescence. Urinary scent marks was similarly elevated by sham and TBI mice in response to a female stimulus compared to the empty open field (a). The ability to deposit scent marks was not affected by injury-related changes in anxiety or bladder structure, as sham and TBI mice spent a similar amount of time in the center of the open field (b) and exhibited similar bladder volumes upon autopsy (c). (TIF) [file pone.0103386.s003.tif]
