# Supplementary material for: Sociosexual and Communication Deficits after Traumatic Injury to the Developing Murine Brain
Source: PLoS One. 2014 Aug 8;9(8):e103386. doi: 10.1371/journal.pone.0103386 (PMC4126664; doi:10.1371/journal.pone.0103386)
Supplement: Table S1 — Call parameters not affected by injury in either cohort. (DOCX) [file pone.0103386.s004.docx]

Supplementary Table 1: Call parameters not affected by injury in either cohort

|  |  | **Median Frequency (kHz)** | | |
| --- | --- | --- | --- | --- |
|  |  | Sham | TBI | p-value |
| **Cohort 1** | female | 79.48 ± 1.87 | 82.52 ± 2.34 | 0.338 |
|  | female bedding | 77.89 ± 1.94 | 80.31 ± 2.24 | 0.444 |
|  | male | 83.04 ± 2.86 | 78.71 ± 3.28 | 0.356 |
| **Cohort 2** | female | 78.14 ± 1.67 | 78.13 ± 0.10 | 0.998 |
|  | female bedding | 76.52 ± 1.71 | 76.53 ± 0.92 | 0.996 |
|  | male | 79.27 ± 1.84 | 81.90 ± 2.58 | 0.356 |
|  | | **Latency to first call (sec)** | | |
|  |  | Sham | TBI | p-value |
| **Cohort 1** | female | 4.13 ± 0.78 | 2.42 ± 0.79 | 0.160 |
|  | female bedding | 1.96 ± 0.61 | 3.50 ± 1.89 | 0.423 |
|  | male | 6.20 ± 1.41 | 3.42 ± 0.63 | 0.128 |
| **Cohort 2** | female | 16.47 ± 2.14 | 18.58 ± 4.61 | 0.653 |
|  | female bedding | 7.21 ± 1.06 | 8.80 ± 1.84 | 0.443 |
|  | male | 35.24 ± 11.76 | 45.31 ± 24.66 | 0.691 |
|  | | **Fraction of calls within bursts** | | |
|  |  | Sham | TBI | p-value |
| **Cohort 1** | female | 0.91 ± 0.02 | 0.96 ± 0.01 | 0.064 |
|  | female bedding | 0.94 ± 0.02 | 0.96 ± 0.00 | 0.354 |
|  | male | 0.76 ± 0.08 | 0.93 ± 0.02 | 0.125 |
| **Cohort 2** | female | 0.96 ± 0.00 | 0.96 ± 0.00 | 0.571 |
|  | female bedding | 0.96 ± 0.01 | 0.91 ± 0.96 | 0.198 |
|  | male | 0.95 ± 0.01 | 0.94 ± 0.02 | 0.791 |
